# Sedation With Propofol Has No Effect on Capsule Endoscopy Completion Rates: A Prospective Single-Center Study

**DOI:** 10.1097/MD.0000000000001140

**Published:** 2015-07-13

**Authors:** Huo-Ye Gan, Yi-Jie Weng, Wei-Guang Qiao, Zhen-Yu Chen, Zhi-Min Xu, Yang Bai, Wei Gong, Tian-Mo Wan, De-Shou Pan, Yong-Sheng Shi, Ai-Jun Qiu, Fa-Chao Zhi

**Affiliations:** From the Department of Gastroenterology, Nanfang Hospital, Southern Medical University, Guangzhou, China (H-YG, Y-JW, W-GQ, Z-YC, Z-MX, YB, WG, T-MW, D-SP, F-CZ); Department of Anesthesiology, Nanfang Hospital, Southern Medical University, Guangzhou, China (Y-SS, A-JQ); and Department of Gastroenterology, Qingyuan City People's Hospital of Jinan University, Guangdong, China (H-YG).

## Abstract

Patients who need both capsule endoscopy (CE) and colonoscopy often undergo both examinations on the same day to avoid repeated bowel preparation and fasting. Sedation can relieve pain and is commonly used for colonoscopies but may influence the CE completion rate.

To determine whether sedation with propofol influences the completion rate and small-bowel transit time (SBTT) of CE.

From July 2014 to December 2014, patients (18–65 years old) who needed both CE and colonoscopy were assessed consecutively for enrollment in our study. Colonoscopies were performed with or without sedation based on patient preferences on the day of capsule ingestion. The completion rate, SBTT, and diagnostic yield of CEs were recorded. Patients’ satisfaction and pain scores were also recorded.

Sedation with propofol had no significant effect on CE completion rates (83.3% sedation group vs 81.8% nonsedation group, *P* = 0.86) but was associated with increased SBTT (403.6 ± 160.3 sedation group vs 334.5 ± 134.4 nonsedation group, *P* = 0.006). The diagnostic yields in the sedation and nonsedation groups were 69.4% and 65.9%, respectively (*P* = 0.74). The median satisfaction scores were 8.6 in the sedation group and 3.5 in the nonsedation group (*P* < 0.001). Median pain scores were 1.4 in the sedation group and 6.7 in the nonsedation group (*P* < 0.001).

Sedation with propofol increased SBTT but had no effect on CE completion rates, suggesting that CE and colonoscopy with propofol can be performed on the same day (clinical trial registration number: ChiCTR-ONRC-14004866).

## INTRODUCTION

Capsule endoscopy (CE) was first introduced by Iddan G. in 2000 as a safe and noninvasive method for recording images of the digestive tract.^1–3^ Advantages of CE include its simplicity and lack of discomfort. Currently, common indications for CE include obscure gastrointestinal bleeding (OGIB), Crohn disease, small bowel tumors, polyps, and malabsorption syndromes,^4–11^ and CE is regarded as one of the most important milestones in gastrointestinal diagnostics since the introduction of endoscopy.^12^

The European Society for Gastrointestinal Endoscopy guidelines on small bowel CE recommend polyethylene glycol (PEG)-based regimens as first-line small bowel preparations before CE. The use of PEG-based regimens is supported by good efficacy and safety data (Grade A).^13^ In some cases, an individual requires both CE and colonoscopy to confirm a diagnosis. This combination has become more common in recent years due to the increasing occurrence of gastrointestinal diseases. Unfortunately, both CE and colonoscopy require bowel preparation, which may occasionally cause discomfort. To avoid repeated bowel preparation and fasting, many patients choose to undergo endoscopy (CEs) and colonoscopy on the same day.

Theoretically, sedation can affect gastrointestinal motility; thus, for patients who need both CEs and colonoscopies on the same day, sedation is not recommended because of its potential to reduce the CE completion rate. However, many patients who undergo colonoscopies without sedation experience significant pain. Sedation is intended to minimize patient discomfort, increase compliance, and in most circumstances, improve the quality of the examination.^14^

Most patients prefer the use of sedation for colonoscopy. In particular, propofol, which has a short elimination half-life, has gained wide acceptance as a drug for sedation and is used extensively.^15^ Sedation with propofol is increasingly used for colonoscopies as it is believed to shorten recovery times.

To evaluate the effect of sedation on CE, we conducted a prospective review of patients who underwent CEs and colonoscopies with or without propofol on the same day.

## METHODS

### Study Design and Patient Population

This was a prospective trial. From July 2014 to December 2014, 18- to 65-year-old patients who underwent CEs and colonoscopies on the same day at Nanfang Hospital (Guangzhou, Guangdong Province, China) were enrolled consecutively. Exclusion criteria included patients with a history of gastric and/or small bowel surgery, obstructive symptoms, ileostomies, diabetes mellitus with evidence of end-organ damage, hypokalemia, use of prokinetic medications within 5 days before the procedure and clinical hyper- or hypothyroidism. Each patient provided written informed consent before beginning the study procedure. Subjects were then assigned to the sedation or nonsedation group based on their preferences. This study was approved by the Institutional Review Board of Nanfang Hospital and registered in the Chinese Clinical Trial Registry (clinical trial registration number: ChiCTR-ONRC-14004866).

### Bowel Preparation

Patients were instructed to eat a soft breakfast on the day before the procedure and to maintain a fluids-only diet starting at lunch time on the day before the procedure. Then, patients were asked to consume 4 L of PEG electrolyte solution (WanHe Pharmaceutical Co., Ltd., Shenzhen, Guangdong Province, China). Specifically, 2 L was consumed between 19:00 and 21:00 on the night before the procedure, and the remaining 2 L was consumed between 3:00 and 5:00 on the day of the procedure. Patients were encouraged to drink more clear liquids following the purgative to ensure adequate hydration and were instructed to take nothing by mouth after 5:00 am on the day of the procedure.

### Procedure

All CE procedures (Intromedic Co. Ltd, Seoul, Korea) commenced between 8:00 am and 9:00 am. At the conclusion of the procedure, patients were asked to take a walk to shorten the gastric transit time. The location of the capsule endoscope was checked by a workstation's real-time monitoring system 2 h later. If the capsule was found to be present in the stomach, upper gastrointestinal endoscopy was performed. Then, a conventional colonoscopy (CFH260AL; Olympus, Tokyo, Japan) was performed before 12:00 am by 1 of 5 attending gastroenterologists, each with more than 10 years of experience. In the sedation group, each patient received a continuous gravity-regulated infusion of intravenous normal saline. Before sedation, all patients received supplemental oxygen (2 L/min) by nasal cannula. An electrocardiogram was obtained, and pulse oximetry, heart rate, and blood pressure were monitored. During endoscopy, patients with spontaneous breathing were sedated only with an initial bolus of propofol (1.5 mg/kg body weight, intravenously). Sedation was maintained with repeated doses of 10 to 20 mg propofol during insertion of the colonoscope by anesthesiologists. Generally, no additional propofol was administered during the withdrawal phase.

### Data Collection

Patient data, including demographics, age, sex, and body mass index (BMI), were recorded. The primary endpoints of the study were CE completion rate and the small-bowel transit time (SBTT). Secondary endpoints were diagnostic yield and adverse events. All CE videos were reviewed by 2 experienced gastroenterologists who were blinded to the group assignment. The completion rate was defined as the frequency of CE that reached the cecum within the life of the battery, and SBTT was defined as the elapsed time from the first duodenal image to the first cecal image. An incomplete examination was defined by the absence of a large-bowel image at the completion of the CE recording. The SBTT was censored when the last image was a small-bowel image in cases of an incomplete examination, which indicated that the battery power had expired before the camera entered the cecum. A positive CE was defined as any abnormal finding in the small bowel. Capsule retention was defined as the presence of the capsule in the gastrointestinal tract for at least 2 weeks after ingestion. The following events were considered adverse events: perforation, oxygen saturation under 85% for over 30 s, heart rate under 40 per min, and blood pressure <80/50 mm Hg, the need for mechanical ventilation, or any cardiorespiratory event requiring an anesthesiologist's assistance. Approximately an hour after the procedure, all patients completed a questionnaire regarding their willingness to undergo a similar examination and rated their satisfaction and pain level according to a standard 10-cm visual analog scale. The visual analog scale consisted of a 10-cm line scaled from 0 (no pain) to 10 (very painful). Baseline values were also recorded.

### Statistical Analysis

Our sample size (N = 71) was planned before the study, after evaluating previously reported CE completion rates (86.8%).^16^ Calculations were based on the assumptions of α = 0.05, 1 – β = 0.80 and an expected between-groups difference of 20% or greater,^17^ which was thought to constitute a minimal clinically meaningful difference.

Percentages were used to describe categorical variables; means (standard deviation) or medians (range) were used to describe continuous variables. For categorical variables, associations between 2 groups were evaluated with a chi-squared test (applying Fisher's correction when necessary); for continuous variables, the 2-sample *t* test or the Mann–Whitney *U* test was used. For patients without a complete small-bowel view, the SBTT was censored at the last recorded time. All statistical analyses were performed using the IBM SPSS 18.0 software. All tests were performed with the α-level set to 0.05 (2-tailed).

## RESULTS

**FIGURE 1:**
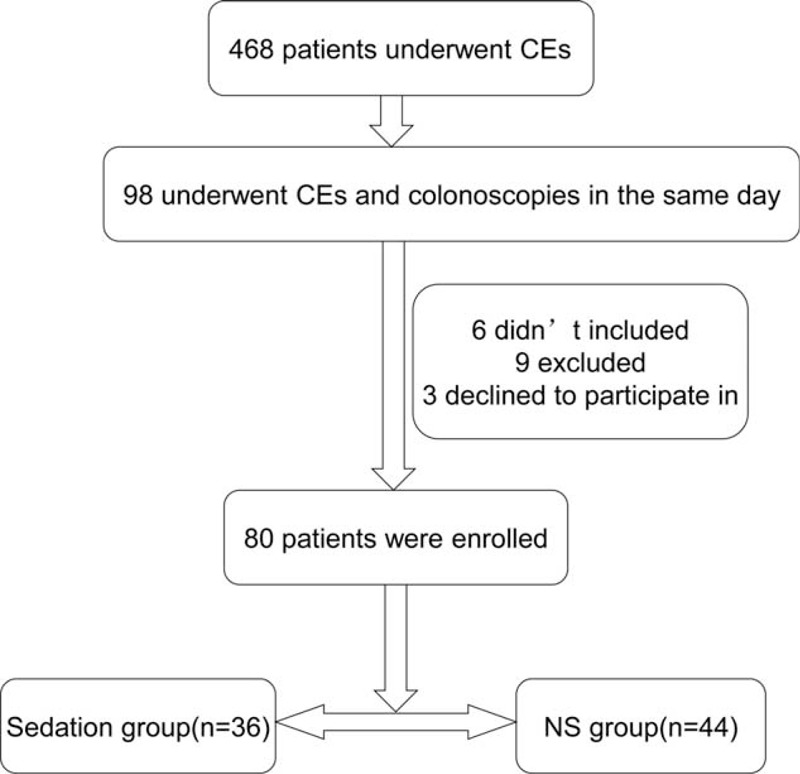
Flow diagram illustrating the study design. CE = capsule endoscopy; NS = nonsedation.

A total of 468 patients underwent CEs between July 2014 and December 2014. Of these patients, 98 underwent CEs and colonoscopies on the same day. Of the 98 patients, 6 were excluded due to age restrictions, 9 were excluded based on additional exclusion criteria, and 3 refused to participate in the study. Therefore, 80 patients were ultimately enrolled, 36 of whom were assigned to the sedation group at their request (Figure 1). Our target sample size was reached.

The baseline characteristics of the 2 groups were not significantly different. The mean ± standard deviation ages of the sedation and nonsedation groups were 45.9 ± 14.2 and 43.3 ± 17.0 years, respectively. The mean ± standard deviation BMIs of the sedation and nonsedation groups were 22.0 ± 3.5 and 22.4 ± 3.8, respectively. Men represented 75.0% (n = 27) of the sedation group and 63.6% (n = 28) of the nonsedation group. The mean ± standard deviation dose of propofol was 155.3 ± 30.0 mg. The most frequent indication for small-bowel CE in the sedation group was abdominal pain with suspected small bowel diseases (n = 9), followed by assessment of abnormal findings on previous results (eg, laboratory tests, scans, and other procedures) (n = 6). Other indications included OGIB (n = 5), inflammatory bowel disease (n = 4), and others (n = 12). In the nonsedation group, the most frequent indication for small bowel CE was also abdominal pain with suspected small bowel diseases (n = 13), but followed by OGIB (n = 12). Other indications included inflammatory bowel disease (n = 5), assessment of abnormal findings on previous results (n = 4), and others (n = 10) (Table 1).

**TABLE 1:**
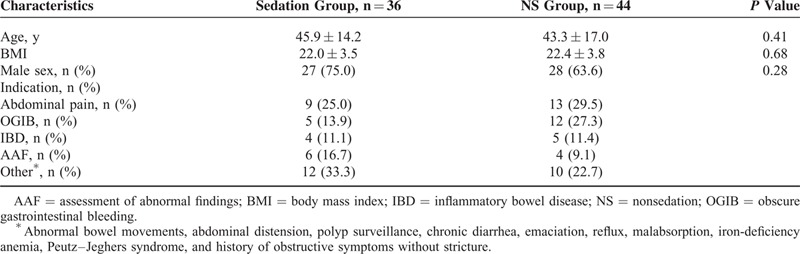
Basic Demographics and Indications for Capsule Endoscopy

There was no difference in the overall CE completion rate between the sedation and nonsedation groups (83.3% [30/36] vs 81.8% [36/44], respectively). In the sedation group, 6 patients were censored for SBTT; 8 were censored in the nonsedation group in cases where the CE battery lost power before the camera entered the cecum. The median SBTT was 403.6 ± 160.3 min in the sedation group and 334.5 ± 134.4 min in the nonsedation group (*P* = 0.006). The median SBTT of the subgroup of patients who completed the CE in the sedation and nonsedation groups were 359.8 ± 125.4 and 299.7 ± 111.1 min, respectively (*P* = 0.007) (Table 2). In the incomplete cases from the sedation and nonsedation groups, the average lifespan of the batteries was 653.6 ± 192.6 and 549.8 ± 122.6 min, respectively (*P* = 0.082).

**TABLE 2:**
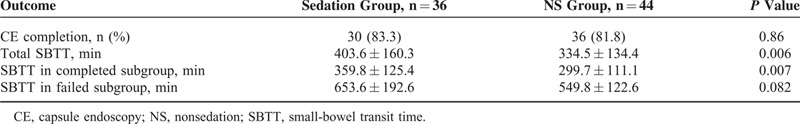
CE Completion Rate and SBTT

Two patients in the sedation group and 3 in the nonsedation group had gastroscopy-assisted CEs. The CE findings are presented in Table 3. The diagnostic yields of the sedation group and the nonsedation group were 69.4% and 65.9%, respectively (*P* = 0.74). The most frequent findings in the sedation group were ulcers (n = 8), followed by erosions (n = 4). Other findings included lymphangiectasia (n = 3), tumors (n = 3), red spots (n = 2), angioectasia (n = 2), active bleeding (n = 1), follicular hyperplasia of the terminal ileum (n = 1), and parasites (n = 1). In the nonsedation group, the most frequent findings were also ulcers (n = 8), followed by lymphangiectasia (n = 5). Other findings included tumors (n = 4), red spots (n = 3), active bleeding (n = 3), erosions (n = 2), angioectasia (n = 2), follicular hyperplasia of the terminal ileum (n = 1), and parasite (n = 1).

**TABLE 3:**
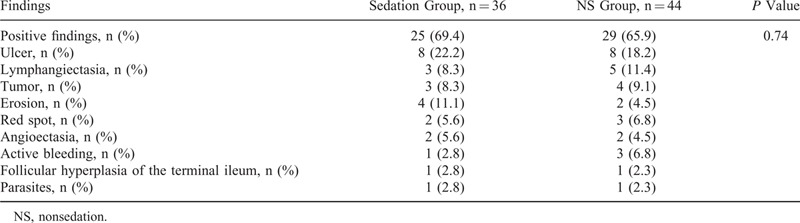
Findings of Capsule Endoscopies in the Sedation and Nonsedation Groups

No CE device was retained in the gastrointestinal tract during the study. Only 1 patient in the sedation group suffered transient oxygen desaturation (<85% oxygen saturation for longer than 30 s), which was addressed by increasing the oxygen flow rate and performing a jaw thrust maneuver. No patient required mechanical ventilation or endotracheal intubation. There were no cases of colon perforation during the study.

All patients completed the postprocedure questionnaire. Median satisfaction scores were 8.6 in the sedation group and 3.5 in the nonsedation group (*P* < 0.001). The median pain scores were 1.4 in the sedation group and 6.7 in the nonsedation group (*P* < 0.001) (Table 4). All of the patients in the sedation group and 27.3% of patients in the nonsedation group reported willingness to have another examination performed in the same fashion. All colonoscopies were completed in the sedation group, while 1 patient in the nonsedation group did not complete the colonoscopy because of pain. Nevertheless, there were no statistically significant differences in colonoscopy completion rates between the 2 groups. In the sedation and nonsedation groups, 83.3% and 4.5% of patients (satisfaction scores ≥8), respectively, were satisfied with the procedure (*P* < 0.001).

**TABLE 4:**
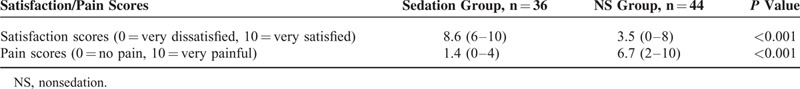
Satisfaction Scores and Pain Scores for Patients in Both Groups, Presented as Median (Range)

## DISCUSSION

In some areas (such as China), medical resources are unevenly distributed. Occasionally, patients (eg, patients with suspected Crohn disease, negative ileocolonoscopy findings, and absence of obstructive symptoms or known stenosis; patients with OGIB; patients with iron-deficiency anemia; patients with suspected small-bowel tumors; patients with unexplained chronic diarrhea; or patients with Peutz–Jeghers syndrome) with unexplainable results are referred from local hospitals to superior medical institutions. Although those patients might have already undergone a colonoscopy at local hospital, physicians at the superior medical institution might advise them to undergo both CE and colonoscopies (considering that physicians in local hospital may lack clinical experience). In addition, patients with established Crohn disease may require both CE and colonoscopies during the course of the disease.

The European Society for Gastrointestinal Endoscopy recommended bowel preparation before CE for better efficacy.^13^ Many patients require CE or colonoscopy, both of which require bowel preparation. A large number of patients choose to undergo CE and colonoscopy on the same day. However, colonoscopy without sedation generally causes pain; thus, patients typically prefer to receive sedation when undergoing a colonoscopy. In some countries, such as China, the total cost of sedation has generally been reasonable (approximately 80 dollars),^18^ and most patients have accepted it. There is a concern, however, that sedation may affect gastrointestinal motility and the completion rate of CE. Indeed, few studies have examined the effect of sedation with propofol on CE, and little is known about the influence of propofol on gastrointestinal motility. Therefore, we conducted a prospective review of patients who underwent same-day CE and colonoscopy with or without sedation.

First, in our prospective analysis of the use of sedation with propofol on CE, we demonstrated that sedation with propofol had no effect on the CE completion rate. Specifically, no differences were detected in the overall completion rates of CE between the 2 groups (*P* = 0.63). This finding was consistent with previous reports^16,19^ and suggests that sedation with propofol had no effect on CE completion rates. Furthermore, no CE devices were observed when performing colonoscopies.

In addition to this finding, our analyses showed that sedation with propofol increased SBTT. Three previous studies have documented a mean SBTT ranging from 260.8 to 297 min, similar to the nonsedation group in the present study.^16,20,21^ However, SBTT in the sedation group in our study was longer than in the nonsedation group, possibly because propofol may slow transit through the small bowel.

Again, although sedation with propofol did prolong the SBTT, it had no effect on completion rate, as CE completion time generally did not extend beyond the lifespan of the battery (8–12 h).

Moreover, the overall diagnostic yield of CE was not significantly different between the sedation and nonsedation groups and was similar to previous studies, ranging from 53.4% to 65.9%.^16,21,22^ Therefore, although sedation increased SBTT significantly, the diagnostic yield was similar regardless of whether sedation was used. Overall, then, the present study shows that propofol can be safely and effectively used for endoscopic procedures and is associated with by high rates of patient satisfaction, few side effects, and a high degree of patient acceptance.

As we know, performing the CE first has less effect on the gastric transit time when CE and colonoscopy with propofol take place on the same day. To minimize the effect on gastric transit time, we suggest that patients undergo CE first.

Our study does have limitations related to the fact that it was a small, single-center, nonrandomized controlled study. Furthermore, SBTT could not be accurately measured in cases of incomplete CE. We chose to censor the SBTT at the last small bowel image instead of excluding patients with incomplete CE from SBTT comparisons. This ensured that incomplete cases were represented in the SBTT comparisons for both groups. Given that the CE completion rates were similar in the 2 study groups, we believe that this approach helped to account for potential bias. The maximum SBTT values in the incomplete cases from the sedation and nonsedation groups were 909 and 1133 min, respectively.

Our research aimed to increase awareness and knowledge of the use of sedation with propofol during CE. Additional large multicenter prospective studies must be conducted to further assess the effect of sedation with propofol on outcomes among patients who undergo CEs and colonoscopies on the same day. We are also looking forward to the development of new anesthetics that will have minimal effects on CE completion rates and SBTT.

In conclusion, when CE and colonoscopy were performed on the same day, the use of sedation with propofol had no effect on CE completion rates and alleviated patients’ suffering.
